# Angiography-based coronary microvascular assessment with and without intracoronary pressure measurements: a systematic review

**DOI:** 10.1007/s00392-023-02338-6

**Published:** 2023-11-21

**Authors:** Michael Kest, András Ágoston, Gábor Tamás Szabó, Attila Kiss, Áron Üveges, Dániel Czuriga, András Komócsi, István Hizoh, Zsolt Kőszegi

**Affiliations:** 1 Szabolcs-Szatmár-Bereg County Hospitals and University Teaching Hospital, Nyíregyháza, Hungary; 2 Division of Cardiology, Department of Cardiology, Faculty of Medicine, University of Debrecen, Debrecen, Hungary; 3 Kálmán Laki Doctoral School of Biomedical and Clinical Sciences, University of Debrecen, Debrecen, Hungary; 4 Center for Biomedical Research and Translational Surgery, Medical University Vienna, Vienna, Austria; 5 Heart Institute, Medical School, University of Pécs, Pécs, Hungary; 6 Heart and Vascular Center, Semmelweis University, Budapest, Hungary

**Keywords:** Coronary microvascular dysfunction, Coronary microvascular assessment, Index of microvascular resistance, Angiographic microvascular assessment, Coronary blood flow

## Abstract

**Background:**

In recent years, several indices have been proposed for quantifying coronary microvascular resistance. We intended to conduct a comprehensive review that systematically evaluates indices of microvascular resistance derived from angiography.

**Objective:**

The objective of this study was to identify and analyze angiography-derived indices of microvascular resistance that have been validated against an invasive reference method. We aimed to compare their limits of agreement with their reference methods and explore their advantages and inherent limitations.

**Methods and results:**

We searched PubMed from inception until 2022 for studies on different techniques for quantifying microvascular resistance. Seven studies met the inclusion criteria. Five studies included techniques that applied calculations based solely on invasive angiography, and were validated against invasively measured thermodilution-derived index of microvascular resistance. The remaining two studies combined angiography with invasively measured intracoronary pressure data, and were validated against invasive Doppler measurements. We converted the ± 1.96 standard deviation limits of agreement with the reference method from the seven studies into percentages relative to the cut-off value of the reference method. The lower limits of agreement for angiography-based methods ranged from − 122 to − 60%, while the upper limits ranged from 74 to 135%. The range of the limits of agreement was considerably lower for the two combined angiography- and pressure-based methods, standing at − 52 to 60% and − 25 to 27%.

**Conclusion:**

Our findings suggest that combined angiography- and pressure-based methods provide a more reliable assessment of microvascular resistance compared to methods relying solely on angiography.

**Graphical Abstract:**

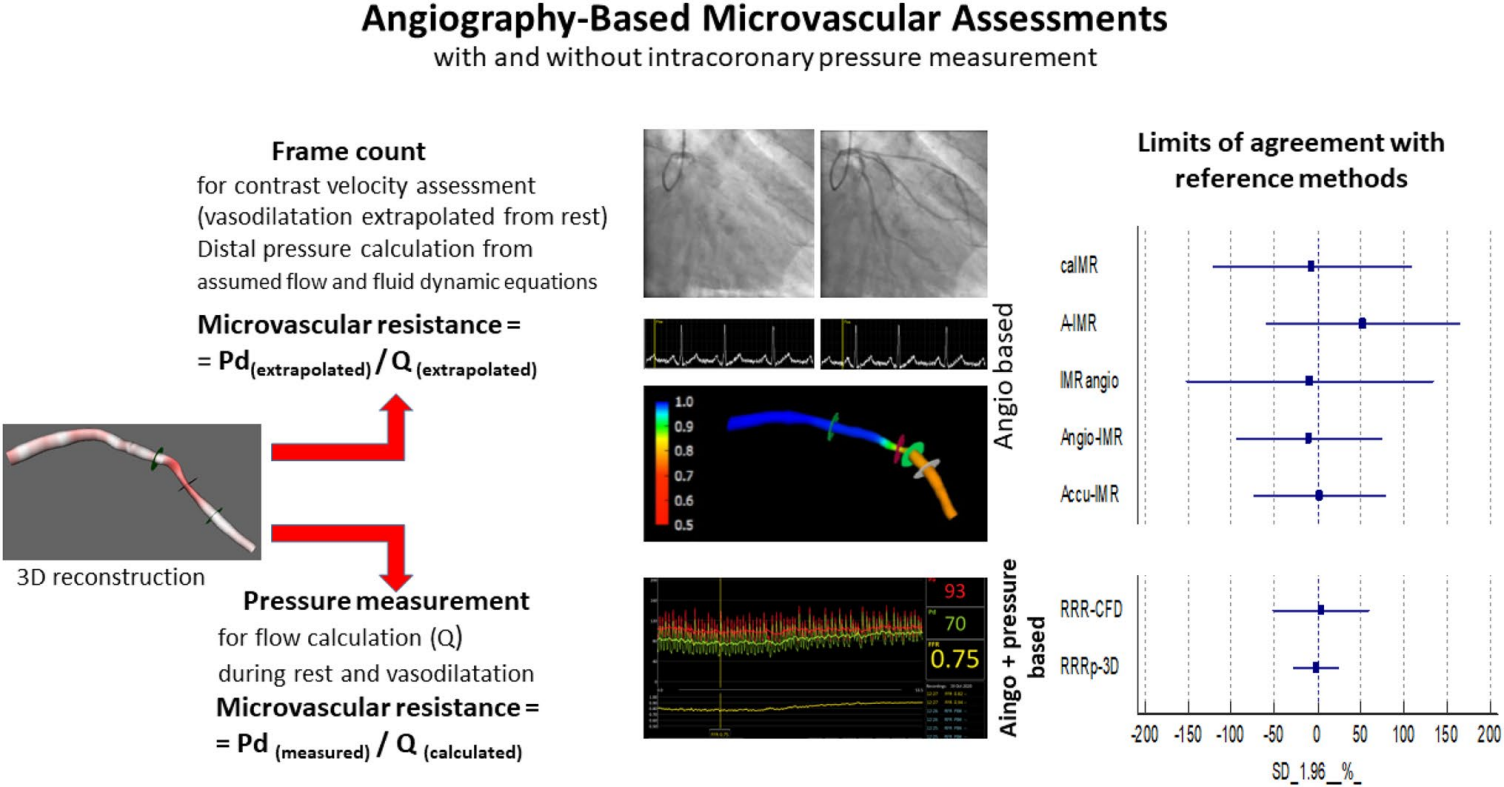

Central illustration. Comparative assessment of image-based methods quantifying microvascular resistance with and without intracoronary pressure measurements. Angiography-based methods rely on angiography alone to calculate the microvascular resistance by utilizing angiographic frame counting to extrapolate coronary flow (*Q*) and subsequently deriving distal coronary pressure using fluid dynamic equations. Combined angiography- and pressure-based methods utilize invasive intracoronary pressure gradients measured during rest and maximal vasodilation to determine coronary flow in their calculation of microvascular resistance. The combined methods showed more acceptable levels of agreement with their reference methods compared to angiography-based methods alone.

## Introduction

Ischemia with Non-Obstructive Coronary Arteries (INOCA) is a heterogenous condition associated with an impaired quality of life and an increased risk for long-term major adverse cardiac events (MACE) [1–3]. Coronary microvascular dysfunction (CMD) is a subtype of INOCA that can lead to microvascular angina and potentially trigger acute myocardial infarction with non-obstructive coronary arteries (MINOCA) [4].

CMD can be identified by assessing coronary blood flow through the measurement of coronary flow reserve (CFR) or changes in microcirculatory resistance that govern alterations to flow. Coronary microvascular resistance is defined as the ratio of distal coronary pressure (Pd) and distal coronary flow rate (Q) during resting and hyperemic conditions [5].

Diagnostic thresholds for direct assessment of coronary blood flow are typically based on invasive Doppler wire measurements, but these methods are technically demanding and not widely available [6]. Other surrogates of flow, including hyperemic mean transit time (assessed with the bolus thermodilution technique) and absolute coronary flow (assessed with continuous thermodilution), have been proposed to calculate the index of microvascular resistance (IMR) and microvascular resistance reserve (MRR), respectively [7, 8]. Microvascular resistance can also be represented by the resistive reserve ratio (RRR), which is defined as the ratio between basal and hyperemic microvascular resistance (bMR/hMR) [43, 44].

IMR is the product of distal coronary pressure at maximal hyperemia and hyperemic mean transit time and is regarded as the tool of choice for diagnosing CMD, with a value of ≥ 25 units indicating abnormal microcirculatory function [9–11]. However, IMR measurements are subject to various limitations, such as the sensor's location in the vessel, the size of the myocardial territory supplied by the target vessel, and the effect of the operator's manual injection technique on the achieved volumetric saline flow rate [12, 13]. These limitations may account for the inconsistency in normal and pathological IMR values and contribute to the lack of widespread adoption of IMR in clinical practice [14].

To provide a less invasive and more streamlined assessment of the coronary microcirculation, several coronary angiography-derived indices of microcirculatory resistance have recently emerged for assessing the coronary microcirculation without the need for adenosine administration or the use of a pressure wire [15]. These techniques rely on angiographic analysis to extrapolate the coronary flow velocity or the mean transit time (Tmn), while deriving distal pressure using computational fluid dynamics (CFD) or contrast quantitative flow reserve (cQFR). More novel approaches attempt to estimate microvascular resistance by deriving coronary flow from invasive intracoronary pressure gradients measured with standard pressure wires.

This review aims to outline the advantages and limitations of angiography-based microvascular parameters and compare their diagnostic accuracy based on limits of agreement with their respective reference methods.

## Methods

**Fig. 1:**
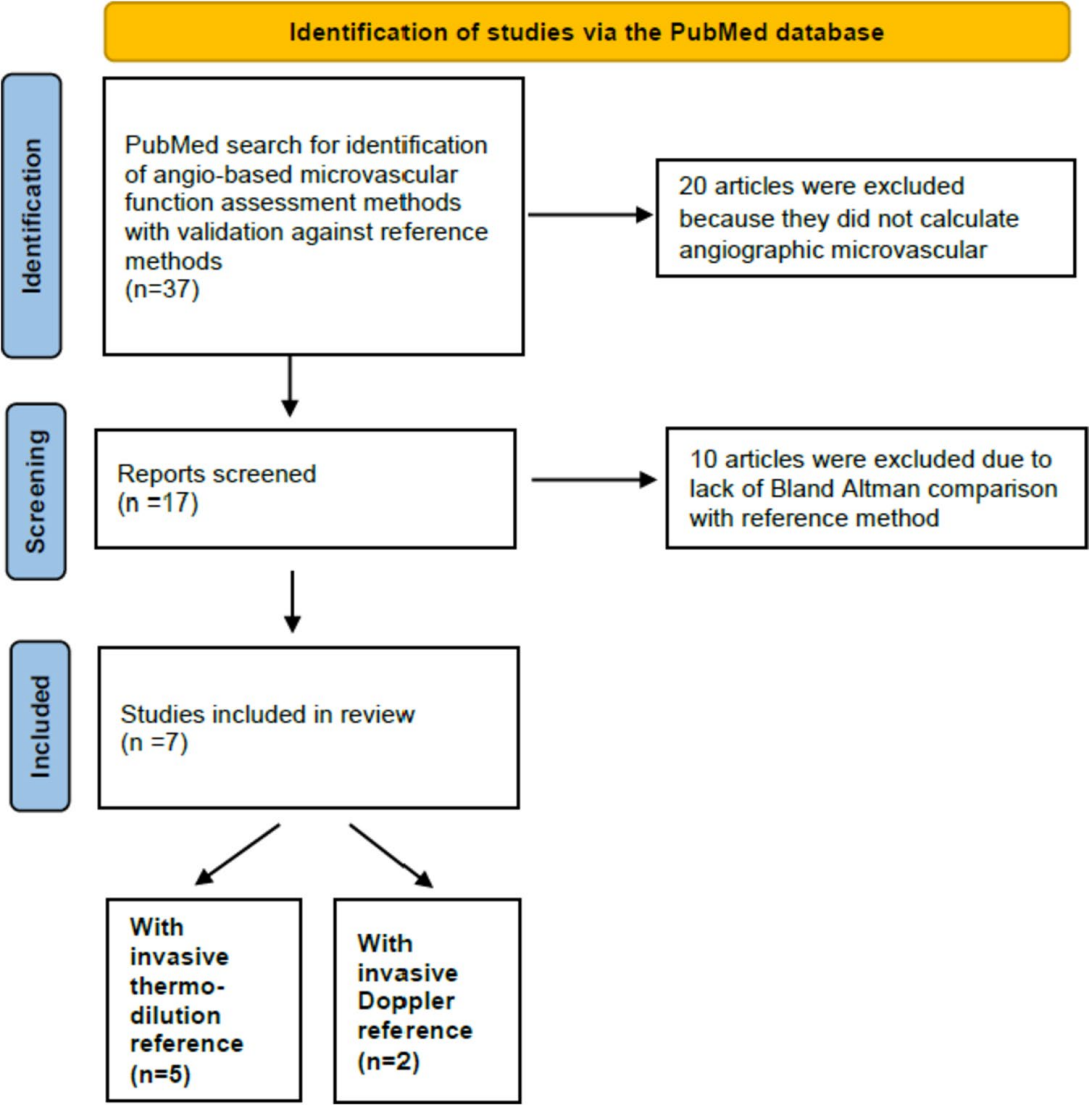
Study selection from the PubMed database. Inclusion and exclusion criteria are detailed. Only studies with Bland Altman analysis against a reference method were considered

A PubMed search was conducted to identify angiography-based techniques that calculate the coronary microvascular resistance with validation against a reference method using the following search algorithm (Fig. 1):

((“IMRAngio”[All Fields] OR “pressure wire free”[All Fields] OR “angiographic”[All Fields]) AND (“index of microcirculatory resistance”[All Fields] OR “microcirculatory resistance”[All Fields]) AND (“IMR”[All Fields] OR “Doppler”[All Fields])) OR (“intracoronary pressure”[All Fields] AND (“computational fluid dynamics”[All Fields] OR “calculations”[All Fields]) AND (“IMR”[All Fields] OR “Intracoronary Doppler”[All Fields])).

**Fig. 2:**
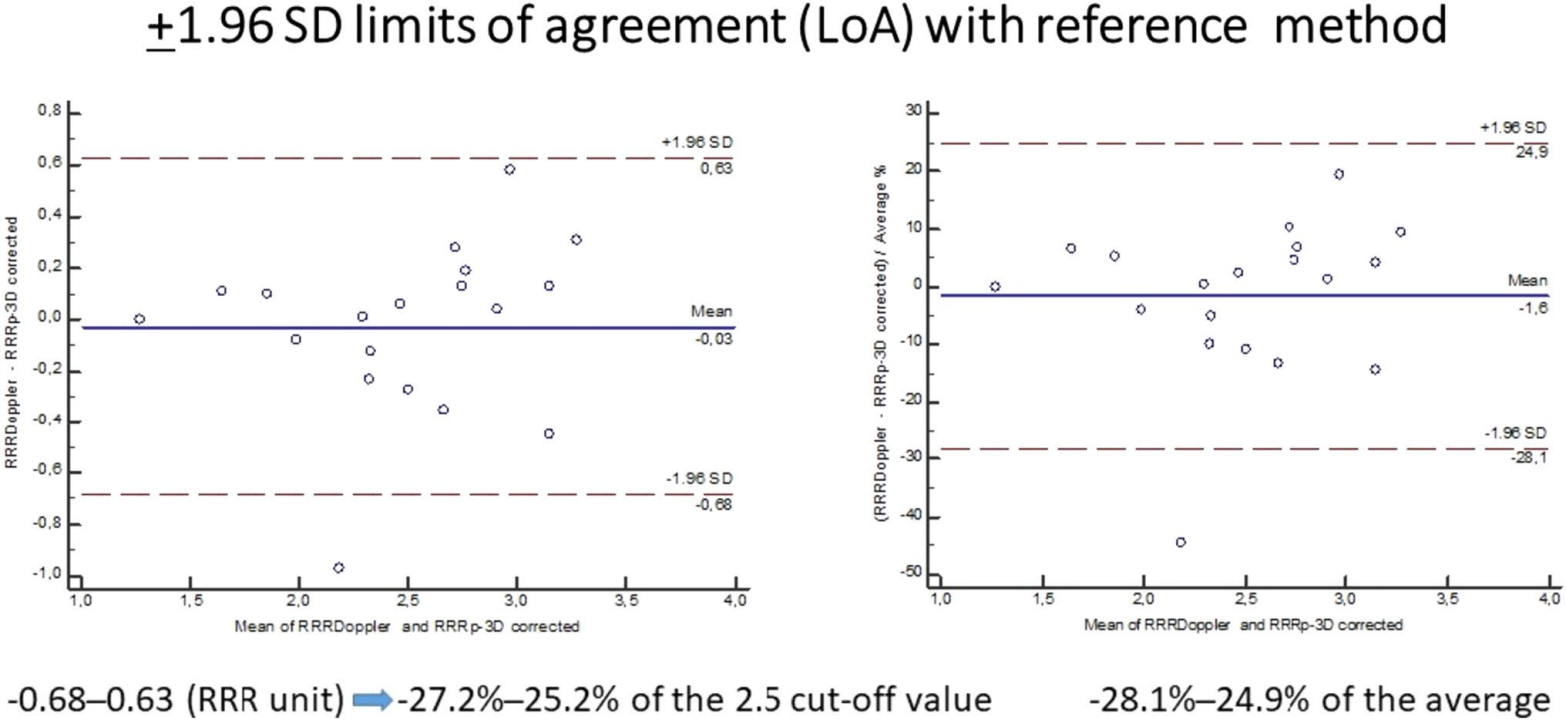
Bland–Altman plot comparing the ± 1.96 limits of agreement of RRRDoppler with RRRP-3D presented as a percentage. The left panel shows a Bland–Altman plot comparing the RRR values derived from reference-standard Doppler measurements (RRR_Doppler_) to the pressure- and 3D-derived RRR values (RRR_P-3D_) which have been corrected for hydrostatic pressure [22]. The plot displays the differences between the two techniques plotted against the averages of the two techniques. The solid horizontal line represents the mean difference (− 0.03), and the dashed lines indicate the limits of agreement (LoA). The LoA are determined as the mean difference minus and plus 1.96 times the standard deviation of the differences (− 0.68 to 0.63). To facilitate interpretation, these LoA values are also expressed as percentages in relation to the cut-off value of RRR (2.5). The right panel demonstrates the calculation of the LoA for the same variables using the Medcalc statistical program. This computation requires the entire dataset from the study to be correlated with the global average of all measurements. The difference between the LoA percentage values obtained from the two approaches is minimal, with only a 0.9% and 0.3% difference. This consistency suggests that the similar results between the two statistical approaches are likely to be replicated in the Bland–Altman plots of the other studies included in this review

Only studies that compared their results with the reference method using Bland–Altman analysis were included in this review. The ± 1.96 standard deviation limits of agreement from the studies were converted to a percentage representing the degree of deviation between the investigated method and the established cut-off value of the reference method. A sample calculation of the limits of agreement for the RRR_P-3D_ index by Tar et al. is demonstrated in Fig. 2.

A critical evaluation of the included studies was then performed based on the approaches used to estimate distal coronary pressure and flow. Of note, the different methods for calculating microvascular resistance identified in this review had different reference methods, namely invasive IMR and Doppler-derived IMR. Consequently, our critical analysis was supported by assessing the percentage deviation between the investigated method and the reference method.

## Results

After conducting a PubMed search, a total of 37 publications were identified. Among these, 20 articles were excluded because they did not calculate the angiographic microvascular reserve. 10 articles were further excluded due to a lack of Bland–Altman comparison with the reference method. This left a total of 7 articles that were included in the review and are listed in Tables 1 and 2. Out of these, 5 articles described indices of microvascular resistance that were based only on angiography (hereinafter referred to as angiography-based methods) and were validated against invasive thermodilution-derived IMR [16–20] (Table 1).

**Table 1:** Angiography-based techniques for microcirculation assessment

Angiography-Based Microvascular assessment								
Index of Microvascular Function	Flow determination/surrogate	Pressure determination/surrogate	Abnormal values	Requirements	± Limits of agreement with reference method (%)	Reference method	Patient population	Possible causes of discordance with reference method
caIMR Author: Ai et al.	Diastolic flow velocity (*V*_diastole_) by resting frame counts and diastolic contrast passing length Hyperemic flow velocity (*V*_hyp_) is assumed to be proportional to V_diastole._multiplied by a constant (*K* = 2.1) Unit: (cm/s)	Pressure drop calculated by computational fluid dynamics	≥ 25	FlashAngio system (including console and separate pressure transducer) (Rainmed Ltd., Suzhou, China)	− 30.7 to 27.4 (− 122% to + 109%)	Invasive IMR by bolus thermodilution	56 patients with CCS and UA	Hyperemic flow velocity is assumed to be proportional to diastolic velocity but may deviate from the actual velocity in patients.
A-IMR Author: Tebaldi et al.	Tmn = vessel length/flow velocity by resting frame counts Unit: (s)	Pd = Pa × cQFR	> 44.2	QAngio^®^ XA 3D software (Medis, Leiden, the Netherlands)	− 26.5 to 73.1 (− 60% to + 165%)	Invasive IMR by bolus thermodilution	44 patients with CCS	Resting flow velocity is significantly lower than hyperemic velocity
Angio-IMR Author: Mejia-Renteria et al.	Tmn = vessel length / flow velocity by “hyperemic” frame counts extrapolated from resting frame counts Unit: (s)	Pd = Pa × cQFR	≥ 25	QAngio^®^ XA 3D software (Medis, Leiden, the Netherlands)	− 23.5 to 18.4 (− 94% to 74%)	Invasive IMR by bolus thermodilution	104 patients	Resting flow velocity is significantly lower than hyperemic velocity
IMR_angio_ Author: De Maria et al.	Tmn = vessel length / flow velocity by hyperemic frame counts Unit: (s)	Pd = Pa × cQFR	≥ 25 (> 40 in STEMI)	QAngio^®^ XA 3D software (Medis, Leiden, the Netherlands)	− 38.2 to 33.6 (− 152% to 134%)	Invasive IMR by bolus thermodilution	145 patients (STEMI = 66; NSTEMI = 43; CCS = 36)	The contrast transport time significantly depends on the timing of contrast injection
AccuIMR Author: Jiang et al.	Tmn = Vessel length / flow velocity by hyperemic frame counts Unit: (s)	Pressure drop calculated by computational fluid dynamics	≥ 25	AccuIMR version 1.0 software (ArteryFlow Technology, Hangzhou, China)	− 18.6 to 19.8 (− 74% to 79%)	Invasive IMR by bolus thermodilution	203 patients	The contrast transport time significantly depends on the timing of contrast injection

*caIMR* coronary angiography-derived index of microvascular resistance, *A-IMR* angio-based index of microcirculatory resistance, *Angio-IMR* wire- and adenosine-free microcirculatory resistive index, *IMRangio* angiography-derived index of microcirculatory resistance, *Tmn* mean transit time, *Pa* proximal pressure, *Pd* distal coronary pressure, *cQFR* contrast quantitative flow ratio, *IMR* index of microcirculatory resistance, *CFR* coronary flow reserve, *CCS* chronic coronary syndrome, *UA* unstable angina, *STEMI* ST-segment elevation myocardial infarction

**Table 2:** Angiography- and pressure-based microvascular assessment.

Index of Microvascular Function	Flow determination/surrogate	Pressure determination/surrogate	Abnormal values	Requirements	± Limits of agreement with reference method (%)	Reference method	Patient population	Possible causes of discordance with reference method
RRR_CFD_ (MVR_CFD hyper_/MVR_CFD rest_) Author: Morris et al.	Flow is calculated from invasively measured intracoronary pressure gradients by computational fluid dynamics model Unit: (ml/s)	Pressure wire (mmHg)	< 2.5	ANSYS (PA, USA) CFD software is required for the off-line calculation	− 1.29 to 1.49 (− 52 to 60%)	Doppler CFR	18 patients with CCS	The measured pressure values were used as boundary conditions in the CFD simulation without hydrostatic pressure correction. This error significantly affects the calculated driving pressure gradient
RRR_p-3D_ Author: Tar et al.	Flow is calculated from invasively measured intracoronary pressure gradients by classic flow equations Unit: (ml/s)	Pressure wire (mmHg), Pd corrected for hydrostatic pressure	< 2.5	Any 3D reconstruction software is appropriate. Hydrostatic pressure calculation can also be performed on 2D images from the lateral view	− 0.68 to 0.63 (− 27 to 25%)	Doppler RRR	17 patients with CCS	The fluid dynamic equations used for the calculation of a single lesion model may oversimplify the actual flow conditions throughout the vessel

*CFD* computational fluid dynamics, *RRR*_*CFD*_ CFD-derived resistance reserve ratio calculated from basal and hyperemic MVR_CFD_ values, *MVR*_*CFD*_ microvascular resistance calculation by computational fluid dynamics, *RRR*_*p-3D*_ pressure- and 3D-derived resistive reserve ratio, *Pd* distal coronary pressure, *CCS* chronic coronary syndrome

The other two articles utilized a combined approach involving the integration of invasive pressure data with angiographic data acquisition (hereinafter referred to as angiography- and pressure-based methods) and were validated against invasive Doppler-based reference methods [21, 22] (Table 2).

### Angiography-based methods to quantify microvascular resistance

The angiography-based methods outlined in this review attempt to recreate the IMR formula by calculating flow and pressure values from angiographic data alone. Ai et al*.* proposed the coronary angiography-derived index of microvascular resistance (caIMR) as the product of hyperemic myocardial resistance (HMR = Pd_hyp_/*V*_hyp_) and a length constant (*L*) mimicking the target vessel length in which contrast was passed from its inlet to the distal segment [16]. Pd_hyp_ is calculated from computational fluid dynamics (CFD) simulations, while *V*_hyp_ is assumed to be proportional to diastolic flow velocity (*V*_diastole_) multiplied by a constant (*K* = 2.1). *V*_diastole_ is extrapolated from resting frame counts and contrast travel length in diastole using an adjusted form of the TIMI frame count method. In 56 patients with chronic coronary syndrome (CCS) and unstable angina (UA), caIMR showed a discordance of − 122 to + 109% from the established cut-off value of 25, with ± 1.96 SD limits of agreement ranging from − 30.7 to 27.4 compared to invasive IMR [16].

Other angiography-based methods derived distal pressure from the product of proximal aortic pressure and contrast quantitative flow ratio (Pd = Pa × cQFR). cQFR allows for the computation of fractional flow reserve (FFR) pressure ratios (Pd/Pa) based on 3D quantitative coronary angiography [23]. Its calculation requires the extrapolation or modeling of hyperemic flow velocity from the resting TIMI frame count based on data derived from previous studies [23, 24]. By calculating the Tmn from the ratio of vessel length corresponding to the number of frames for contrast dye to travel from the guiding catheter to a distal reference divided by flow velocity extrapolated from frame counting, angiography-derived IMR could be represented as [Pa × cQFR × (vessel length/flow velocity)] [25].

The angio-based index of microcirculatory resistance (A-IMR) proposed by Tebaldi et al*.* relies on resting frame counts and had ± 1.96 SD limits of agreement with invasive IMR of − 26.5 to 73.1 (− 60 to + 165%) in 44 patients with CCS [17]. Mejia-Renteria et al*.* avoided the use of a hyperemic agent by extrapolating “hyperemic” frame counts from resting frame counts [19]. Their index, Angio-IMR, was proposed as (Pa_rest_ – [0.1 × Pa_rest_]) × QFR × (vessel length/*V*_hyp_), where *V*_hyp_ is the hyperemic coronary flow velocity extrapolated from resting contrast velocity using a quadratic function on the basis of a given database. The limits of agreement with invasive IMR were − 23.5 to 18.4 (− 94 to + 74%) compared to the cut-off value of 25 in 104 patients [19]. De Maria et al. used hyperemic frame counts to calculate the flow velocity in their equation for IMR_angio_ in patients with STEMI (*N* = 66) [18], NSTEMI (*N* = 43), and CCS (*N* = 36) [26]. The combined ± 1.96 SD limits of agreement with invasive IMR in both studies was − 38.2 to 33.6 (− 152 to 134%) compared to the cut-off values of 40 in STEMI and 25 in NSTEMI and CCS patients. Jiang et al. also utilized hyperemic frame counts in their calculation of AccuIMR but calculated the pressure drop by a CFD model instead of cQFR. The limits of agreement with invasive IMR were − 18.6 to 19.8 (− 74 to 79%) in 203 patients [20].

### Combined angiography- and pressure-based methods to quantify microvascular resistance

Morris et al*.* utilized invasively measured intracoronary pressure gradients to calculate absolute volumetric flow using a CFD model [21]. This allowed the subsequent calculation of CFD-derived microvascular resistance (MVR_CFD_), defined as the ratio of wire-derived distal coronary pressure (Pd) and absolute volumetric flow (*Q*_CFD_). The limits of agreement with Doppler-derived resistive reserve ratio (RRR, calculated as the ratio between basal and hyperemic MVR_CFD_) were − 1.29 to 1.49 (− 52 to 60% compared to a cut-off value of 2.5) in 18 patients with chronic coronary syndrome (CCS) [21].

Tar et al. used a similar pressure-based approach to calculate the pressure- and 3D-derived resistive reserve ratio (RRR_p-3D_) by combining classical fluid hemodynamic equations with 3D anatomical parameters and invasive intracoronary pressure data from FFR measurements [22]. In their study, distal coronary pressure was corrected for hydrostatic pressure variations that occur due to the level difference between the catheter tip and pressure wire sensor. The limits of agreement with Doppler-derived RRR in 17 CCS patients were − 0.68 to 0.63 (− 27 to 25% compared to a cut-off value of 2.5).

## Discussion

INOCA is a major cause of chest pain in patients without hemodynamically significant coronary lesions, as assessed by invasive or CT coronary angiography [5]. The associated CMD can also worsen existing hemodynamically significant epicardial coronary disease [27–29]. Patients with INOCA are often misdiagnosed, leading to a negative impact on their physical and mental well-being and an increase in healthcare costs [30]. Abnormalities in the microcirculation have also been implicated in the pathogenesis of several conditions, including apical ballooning (Takotsubo) syndrome [31], hypertension, diabetes, obesity, metabolic syndrome [32], and the cardiovascular manifestations of COVID-19 [33–37]. Therefore, it is essential to establish an appropriate diagnosis to meet the therapeutic needs of patients with INOCA [38].

The increasing recognition of INOCA as a significant cause of ischemic chest pain has led to the development of several indices derived from angiography that measure the microvascular resistance and aid with the assessment of coronary microcirculation. Angiography-based methods calculate the microvascular resistance by estimating coronary flow from angiographic frame counting and subsequently deriving distal coronary pressure using CFD or cQFR. In the case of caIMR, A-IMR and Angio-IMR, resting frame counting was utilized to determine the Tmn value corresponding to coronary flow, whereas both AccuIMR and IMR_angio_ utilized hyperemic frame counting. These indices of microvascular resistance are virtually derived from angiography and lack direct physiological measurements, which could misrepresent the actual state of the microcirculation due to the potential sources of error summarized in Table 1.

In this review, angiography-based methods showed unacceptably high limits of agreement on Bland–Altman analysis. In contrast, combined angiography- and pressure-based methods showed more acceptable levels of agreement. Both RRR_CFD_ and RRR_p-3D_ utilized invasively measured intracoronary pressure gradients to determine coronary flow in their calculation of microvascular resistance. The integration of accurate pressure measurements provides a more physiological basis for the calculations and reduces the risk of bias. This patient- and vessel-specific approach may account for the superior accuracy of these combined methods in assessing the microcirculation.

A recent meta-analysis comprising seven studies found that angiography-derived IMR demonstrated good overall diagnostic accuracy in predicting abnormal invasive IMR, with a sensitivity of 82% and a specificity of 83% [39]. However, Morris et al*.* point out that diagnostic accuracy alone does not reflect the degree of agreement between the two methods and may be imprecise in borderline cases with values close to the cut-off [40]. Instead, a Bland–Altman plot offers a better indication of how accurately angiography-derived IMR agrees with invasive IMR.

### Angiography-based methods to quantify coronary microvascular function (caIMR, A-IMR, Angio-IMR, IMR_angio_)

The angiography-based indices of microvascular resistance identified in this review show wide limits of agreement despite having a reasonable diagnostic performance at identifying abnormal cut-off values in reference to thermodilution-derived IMR.

Table 1 summarizes the ± 1.96 SD limits of agreement, which reflect the potential magnitude of discordance between angiography-based methods and the reference method. Such large discordance can be misleading and may directly impact decision-making in the catheterization laboratory.

The central paradox of adenosine- and pressure wire-free methods is that distal pressure is calculated using fluid dynamic equations that assume hyperemic coronary flow velocity. As summarized in Table 1*,* caIMR relies on diastolic flow to extrapolate hyperemic flow velocity (*V*_hyp_). A-IMR uses resting frame counts to derive resting flow velocity, which in turn is significantly lower than hyperemic velocity. Similarly, Angio-IMR is calculated by extrapolating hyperemic flow from resting flow analysis. In these cases, flow velocity is determined without achieving maximal hyperemia. However, the patient's microvascular function can affect the assumed hyperemic velocity, leading to deviations in calculated QFR values from the patient-specific flow velocities [41, 42]. These deviations can lead to errors affecting equations determining the distal pressure and the resulting error will be multiplied in all subsequent calculations, leading to erroneously large IMR values.

Conversely, IMR_angio_ and AccuIMR rely on hyperemic frame counts to derive flow velocity [18, 20], but this approach may also introduce bias and result in inaccurate Tmn values that deviate from patient-specific values. Although a state of hyperemia theoretically enables more precise detection of microvascular functional abnormalities, reading a hyperemic frame count is challenging due to the difficulty in discerning the contrast wavefront during a high flow rate compared to the resting angiogram. Furthermore, the variability of the detected contrast transport time may be more pronounced during hyperemia as it is heavily influenced by the timing of contrast injection in the cardiac cycle compared to the resting state [42]. This can result in discrepancies between the measured contrast velocity and the actual blood flow velocity within the vessel. In a subsequent study, a non-hyperemic version of IMR_angio_ (NH-IMR_angio_) was proposed, with a cut-off value of > 30 U for detecting abnormal thermodilution-derived IMR in STEMI patients [26]. However, the diagnostic performance of NH-IMR_angio_ was suboptimal in patients with non-ST segment elevation acute coronary syndrome and CCS, possibly due to the inability of a non-hyperemic index to reflect the minimal level of resistance attainable at maximal hyperemia when the microvascular vasodilatory capacity is preserved [26].

### Angiography- and pressure-based methods to quantify coronary microvascular function (MVRCFD, RRR_p-3D_)

The angiography-based techniques discussed in the previous section rely solely on angiography to quantify IMR. In contrast, angiography- and pressure-based methods estimate microvascular resistance by deriving coronary flow from invasive intracoronary pressure gradients measured with standard pressure wires (Table 2).

Morris et al*.* proposed a computational fluid dynamics model to calculate absolute volumetric flow (*Q*_CFD_) from invasive pressure data and 3-D anatomic reconstructions of coronary angiographic images [21]. This enabled the subsequent calculation of MVR_QCFD_ from the ratio of distal pressure (*P*_d_) and *Q*_CFD_.

In this systematic review, the resistance reserve ratio (RRR) was calculated from the basal and hyperemic MVR_QCFD_ values obtained from the Morris et al*.* study to facilitate a direct comparison with the limits of agreement calculated from the study by Tar et al*.* Both studies utilized invasively measured pressure data and compared their results to Doppler-derived RRR.

RRR is an integrated index of microvascular resistance, defined as the ratio between basal and hyperemic microvascular resistance (bMR/hMR) or the ratio of distal coronary pressure (*P*_d_) and distal coronary flow rate (*Q*) during resting and hyperemic conditions [43, 44]. Alternatively, RRR can also be represented as coronary flow reserve (CFR) divided by the ratio between resting and hyperemic distal pressure (*P*_d_). In contrast with IMR, which does not provide information on the vasodilatory capacity of the microcirculation, RRR reliably reflects the ability of the coronary microcirculation to adjust its resistance in response to adenosine and provides prognostic value in both acute myocardial infarction and nonobstructive coronary artery disease [45–47].

Pressure- and 3D-derived CFR (CFR_p−3D_) was proposed by Tar et al. to calculate CFR using invasive intracoronary pressure data and 3D anatomic reconstructions of the target vessel from angiography [22]. Measuring CFR_p−3D_ facilitates the subsequent calculation of the RRR by incorporating distal coronary pressure through the aforementioned formula. Their combined angiography- and pressure-based approach also factored in individual variations in hydrostatic pressure, where distal pressure was corrected for hydrostatic pressure variations caused by the level difference between the tip of the catheter and the pressure wire sensor. RRR_P-3D_ showed a good correlation with Doppler-derived RRR, and better limits of agreement with the Doppler-based method was also reported compared to all methods included in this review. This highlights the importance of correcting distal pressure for variations in hydrostatic pressure to avoid inaccuracies in calculating the driving pressure gradient.

During functional assessment of coronary arteries, hydrostatic pressure variations occur due to the height difference between the pressure sensor and the catheter tip at the vessel orifice in the supine position, where the LAD usually runs upwards while the RCA and LCX run downwards [48]. These variations can influence intracoronary pressure measurements, but their impact has largely been ignored in clinical practice up until recently. In 2019, Kawaguchi et al*.* examined intracoronary pressures in 23 patients and reported significant differences between FFR and resting *P*_d_/*P*_a_ values measured in the supine and prone positions. These differences were mitigated by hydrostatic pressure correction [49]. Üveges et al*.* investigated the effect of hydrostatic pressure on resting *P*_d_/*P*_a_ and FFR based on height differences calculated with 3D coronary reconstruction. In their study, 41 intermediate-severity coronary lesions with FFR values between 0.7 and 0.9 were evaluated and pressure measurements were corrected for height differences by subtracting the hydrostatic pressure gradient from the distal pressure. This correction changed the interpretation of the measurements in 12% and 27% of cases for FFR and resting *P*_d_/*P*_a_, respectively, highlighting the potential clinical significance of hydrostatic pressure measurement [50].

Hydrostatic pressure variations are even more pronounced when invasive pressure data is used to derive coronary flow and subsequently calculate microvascular resistance from the ratio of coronary flow and distal pressure. It is worth noting that, in the study by Morris et al., pressure-derived CFR (CFR_pd_) closely correlated (*R*^2^ 0.92, *P* < 0.001) but systematically underestimated (mean delta − 0.16 ± 0.17) Q_CFD_-derived CFR in their in vivo assessment. In turn, Doppler-derived CFR overestimated CFR_pd_ (mean delta − 0.35 ± 0.46) and a very weak correlation was reported (*R*^2^ 0.32, *P* = 0.1) [21]. We posit that the poor correlation between pressure- and Doppler-derived CFR could be attributed, at least partly, to their lack of correcting the distal coronary pressure (*P*_d_) for variations in hydrostatic pressure, which could significantly impact the calculated driving pressure gradient. In light of the above, the inclusion of invasive pressure data and its correction for hydrostatic pressure in hemodynamic calculations provides a stronger physiological basis for the derived parameters and may help overcome the aforementioned challenges and assumptions of deriving physiology merely from anatomy.

### Clinical implications of combined angiography- and pressure-based microvascular assessment

The 2019 European Society of Cardiology (ESC) guidelines recommend invasive guidewire-based pressure and flow measurements to diagnose a microcirculatory origin of angina in patients with persistent symptoms and either angiographically normal coronary arteries or moderate stenoses with preserved FFR or instantaneous wave-free ratio (iwFR) [9]. Additionally, pharmacological testing with intracoronary ACh injection may be performed to test endothelial function and rule out vasospastic angina or microvascular spasm [9, 51]. The CorMicA trial demonstrated that a tailored treatment strategy based on CFR, IMR, and Ach testing significantly improved angina scores and quality of life in patients with INOCA [52].

Despite these recommendations, invasive microvascular assessment is not widely used due to a lack of consensus on a uniform testing protocol and a general fear of associated complications [53]. Angiography-based methods may facilitate the routine assessment of the coronary microcirculation and help identify underlying pathomechanisms of INOCA, ultimately aiding in the selection of optimal medical therapy [32]. The diagnostic accuracy of these methods could be improved with the inclusion of invasive pressure measurements and accounting for hydrostatic pressure variations during the calculation of distal pressure.

**Fig. 3:**
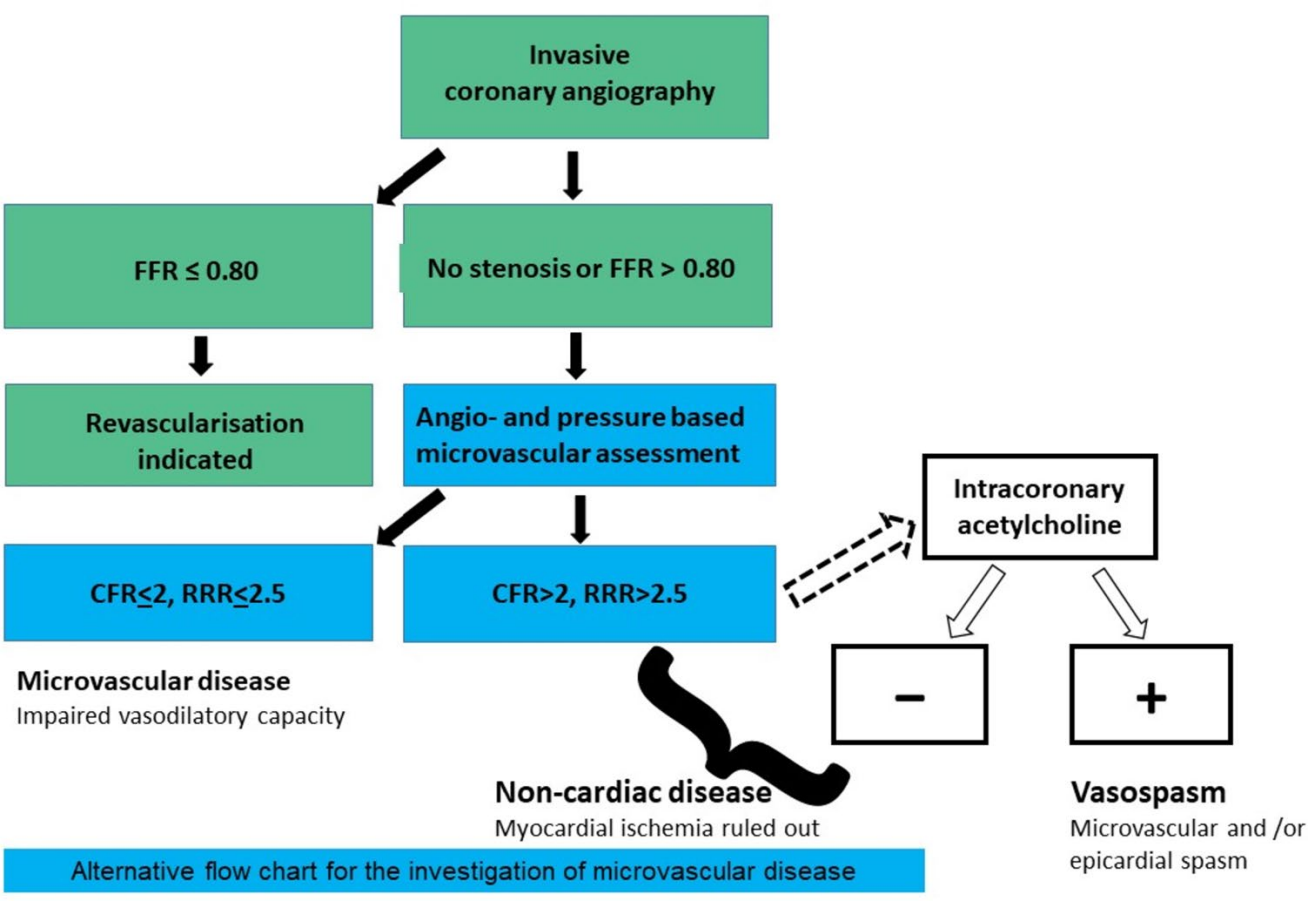
Proposed flow chart for investigating microvascular disease using combined angiography- and pressure-based methods. In cases of epicardial stenosis ranging from 50 to 90% diameter reduction, the initial evaluation of intracoronary pressure gradients (FFR) using a standard pressure enables the differentiation of hemodynamically significant lesions that require revascularization (FFR ≤ 0.80) from those necessitating further investigation to rule out underlying microvascular disease (FFR ≥ 0.80). In patients with persistent symptoms, pressure wire-based microcirculatory resistance measurements could be considered even in the absence of angiographic stenosis. RRR values ≤ 2.5 confirm the existence of micovascular disease, while negative values prompt additional investigation through intracoronary acetylcholine injection to rule out vasospastic angina or exclude a cardiac origin of angina altogether. *FFR* Fractional Flow Reserve, *CFR* Coronary Flow Reserve; *RRR* Resistive Reserve Ratio

Combined angiography- and pressure-based methods in the catheterization laboratory could provide a quick and comprehensive anatomical and functional assessment of both epicardial coronary arteries and the microcirculation. A diagnostic algorithm that incorporates combined angiographic- and pressure-based evaluation of coronary physiology in patients with clinically indicated invasive measurement of FFR is proposed in Fig. 3. Patients without significant epicardial disease (FFR > 0.80 or iwFR > 0.89) could benefit from angiography- and pressure-based evaluation of CFR, RRR, or MRR.

After conducting the proposed investigations, the identification of pathological values suggests the presence of microvascular disease, while patients with normal values may benefit from intracoronary Ach testing to assess micro- and macrovascular reactivity. Accordingly, patients with INOCA can be further classified into those with abnormal vasoconstriction, abnormal vasodilation, or a mixed disease type [1]. In case of negative testing, myocardial ischemia could be ruled out altogether.

## Conclusions

Angiography-based methods for assessing microvascular resistance rely solely on angiography to estimate distal pressure and derive hyperemic flow. However, these methods are limited by their dependence on angiographic frame counting to extrapolate hyperemic flow, and their inability to account for individual variations in microvascular vasodilatory capacity can impact the accuracy of the calculations. In contrast, angiography- and pressure-based methods combine invasively measured pressure gradients with angiographic reconstructions of the target vessel to derive coronary flow. These combined methods show better limits of agreement with their reference methods, particularly when variations in hydrostatic pressure are accounted for. Additionally, coronary flow derived from invasive intracoronary pressure gradients can be routinely obtained during invasive FFR measurement without the need for additional devices or procedures. Subsequent calculation of microvascular resistance from the ratio of distal pressure and pressure-derived coronary flow using these combined methods could streamline the workflow of the comprehensive coronary physiology evaluation recommended by the ESC guidelines (Fig. 3). Further studies are warranted to validate the clinical utility of these combined methods for investigating coronary microvascular dysfunction in various cardiovascular disorders and to establish their efficacy in stratifying patients for individually tailored therapy. Understanding the limitations and potential sources of variability between different methods that assess the microcirculation will enable healthcare providers to make informed decisions about which method is most appropriate for a given patient and thus improve diagnostic accuracy in patients with INOCA. This will ultimately lead to better outcomes and a reduced burden on the healthcare system.

## Limitations

The angiography-based methods and combined angiography- and pressure-based methods identified in this review were compared against different reference methods (invasive IMR and Doppler-derived RRR, respectively). Since the main aim of the study was to determine the diagnostic accuracy of different angiography-derived indices of microvascular resistance using Bland–Altman analysis, the different reference methods used for both groups do not facilitate a straightforward comparison of their limits of agreement. However, both IMR and RRR are indices of microvascular resistance that incorporate pressure and coronary flow (or its surrogate) measurements in their calculations, making a comparison between the two groups feasible. The limits of agreement were presented as a percentage in relation to the cut-off value of the reference method to facilitate the interpretation of the results. If more angiography-derived indices incorporating direct pressure measurements are introduced in the future, a meta-analysis could be conducted with all indices having a unified reference method to verify the results of the current study.

While we acknowledge the value of meta-analytic calculations, their application in this review was hindered by several factors. The limited number of studies and significant methodological differences precluded the assumption of homogeneity, a cornerstone for effective meta-analysis. Also, existing models for cumulative Bland–Altman bias and limits of agreement data differ from those used for effect estimates and 95% CIs, complicating their integration. Despite these limitations, we believe our comprehensive review delivers its main conclusions based on our analysis of the systematically acquired data, and offers valuable insights for future research.
